# Near-complete genome sequence of *Lipomyces tetrasporous* NRRL Y-64009, an oleaginous yeast capable of growing on lignocellulosic hydrolysates

**DOI:** 10.1128/MRA.00426-23

**Published:** 2023-10-31

**Authors:** Sujit Sadashiv Jagtap, Jing-Jing Liu, Hanna E. Walukiewicz, Jasmyn Pangilinan, Anna Lipzen, Steven Ahrendt, Maxim Koriabine, Kelly Cobaugh, Asaf Salamov, Yuko Yoshinaga, Vivian Ng, Chris Daum, Igor V. Grigoriev, Patricia J. Slininger, Bruce S. Dien, Yong-Su Jin, Christopher V. Rao

**Affiliations:** 1 Department of Chemical and Biomolecular Engineering, University of Illinois at Urbana-Champaign, Urbana, Illinois, USA; 2 DOE Center for Advanced Bioenergy and Bioproducts Innovation, University of Illinois at Urbana-Champaign, Urbana, Illinois, USA; 3 US Department of Energy Joint Genome Institute, Lawrence Berkeley National Laboratory, Berkeley, California, USA; 4 Department of Plant and Microbial Biology, University of California Berkeley, Berkeley, California, USA; 5 Bioenergy Research Unit, National Center for Agricultural Utilization Research, USDA-ARS, Peoria, Illinois, USA; 6 Department of Food Science and Nutrition, University of Illinois at Urbana-Champaign, Urbana, Illinois, USA; University of California, Riverside, California, USA

**Keywords:** *Lipomyces*, genome sequence, PacBio sequencing, genome assembly, lipid

## Abstract

*Lipomyces tetrasporous* is an oleaginous yeast that can utilize a variety of plant-based sugars. It accumulates lipids during growth on lignocellulosic biomass hydrolysates. We present the annotated genome sequence of *L. tetrasporous* NRRL Y-64009 to aid in its development as a platform organism for producing lipids and lipid-based bioproducts.

## ANNOUNCEMENT

*Lipomyces tetrasporous* NRRL Y-64009 is an oleaginous yeast with the ability to utilize plant-based sugars, glycerol, starch, and organic acids (1 – 3). Because of its high lipid accumulation (over 60%), this yeast has been considered an appealing host for industrial-scale lipid production (4). It can withstand low pH levels and detoxify inhibitors found in lignocellulosic hydrolysates (5, 6). We sequenced the genome and transcriptome of *L. tetrasporous* NRRL Y-64009 to further investigate its physiology and metabolism to produce biofuels and bioproducts.

The cells were grown for 2 days at 30°C and 250 rpm in YPG medium (10 g/L yeast extract, 20 g/L peptone, and 20 g/L glucose) and harvested OD_600nm_ of 10. Genomic DNA and total RNA were extracted using the Dr. GenTLE (from yeast) High Recovery Kit (Takara Bio Inc., Shiga, Japan) and the RNeasy Mini Kit (Qiagen, Hilden, Germany), respectively (7, 8).

Libraries larger than 10 kb for PacBio sequencing were prepared using 5 ug of genomic DNA (9, 10). The sheared DNA was treated with exonuclease, followed by end repair and blunt adapter ligation with the SMRTbell Template Prep Kit 1.0. Blue Pippin (Sage Science) was used to select the library at an 8-kb cutoff and purified with AMPure PB beads. The prepared SMRTbell template libraries were then sequenced on a Pacific Biosystems Sequel II sequencer with 1 × 900 bp sequencing movie run times using v3 sequencing primer, 8M v1 SMRT cells, and Version 2.0 sequencing chemistry. To remove sequencing artifacts, BBduk and BBMerge from BBTools 36.63 (https://sourceforge.net/projects/bbmap/) were used (11).

The Illumina Truseq Stranded mRNA Library Prep Kit was used to create stranded cDNA libraries (11). For library amplification, 1 ug RNA per sample and eight cycles of PCR were used. The libraries were quantified using a Roche LightCycler 480 real-time PCR instrument and a KAPA Biosystems Next-Generation Sequencing Library qPCR Kit. The flow cell was sequenced on an Illumina NovaSeq sequencer with NovaSeq XP V1 reagent kits and an S4 flow cell. The Illumina NovaSeq S4 was used to build an Illumina library and sequenced 2 × 151. BBDuk (version 38.79) (http://bbtools.jgi.doe.gov) was used to remove contaminants, trim reads containing adapter sequences, and right quality trim reads with quality less than 6. Reads mapped to common contaminants and ribosomal RNA reads using BBMap were removed. Filtered Fastq files were used as input for *de novo* RNA contig assembly. Trinity v2.8.5 was used to assemble consensus sequences from reads that had been filtered and trimmed for quality and contamination (12). Transcript-based gene predictors ESTmap and CombEST were used to incorporate RNA-Seq into the annotation. A genomic mapping and alignment program v2007-local-branch and the BLAST-like alignment tool were used to align RNA-Seq to the genome assembly (13, 14).

The 20.78 Mb genome assembly was composed of 18 contigs (*N*_50_ = 5 Mb), with a sequencing read coverage depth of 267.54× and a GC content of 48.12%. The Joint Genome Institute (JGI) Annotation pipeline (13, 14) was used to predict 8,004 protein-coding genes from the genome.

## Data Availability

MycoCosm (12) provides whole-genome assemblies and annotation https://mycocosm.jgi.doe.gov/Liptet1. JARPMG000000000 is the accession number for this Whole Genome Shotgun project at DDBJ/ENA/GenBank. JARPMG000000000.1 is the version described in this paper. PRJNA928472 and SRR23940483 are the accession numbers for the project and reads, respectively.

## References

[1] Génolevures Consortium, Souciet J-L , Dujon B , Gaillardin C , Johnston M , Baret PV , Cliften P , Sherman DJ , Weissenbach J , Westhof E , Wincker P , Jubin C , Poulain J , Barbe V , Ségurens B , Artiguenave F , Anthouard V , Vacherie B , Val M-E , Fulton RS , Minx P , Wilson R , Durrens P , Jean G , Marck C , Martin T , Nikolski M , Rolland T , Seret M-L , Casarégola S , Despons L , Fairhead C , Fischer G , Lafontaine I , Leh V , Lemaire M , de Montigny J , Neuvéglise C , Thierry A , Blanc-Lenfle I , Bleykasten C , Diffels J , Fritsch E , Frangeul L , Goëffon A , Jauniaux N , Kachouri-Lafond R , Payen C , Potier S , Pribylova L . 2009. Comparative Genomics of Protoploid Saccharomycetaceae. Genome Res 19:1696–1709. doi:10.1101/gr.091546.109 19525356 PMC2765284

[2] Deewan A , Liu J-J , Jagtap SS , Yun EJ , Walukiewicz H , Jin Y-S , Rao CV . 2022. System analysis of Lipomyces starkeyi during growth on various plant-based sugars. Appl Microbiol Biotechnol 106:5629–5642. doi:10.1007/s00253-022-12084-w 35906440

[3] Dien BS , Zhu JY , Slininger PJ , Kurtzman CP , Moser BR , O’Bryan PJ , Gleisner R , Cotta MA . 2016. Conversion of SPORL pretreated douglas fir forest residues into microbial lipids with oleaginous yeasts. RSC Adv 6:20695–20705. doi:10.1039/C5RA24430G

[4] Slininger PJ , Dien BS , Kurtzman CP , Moser BR , Bakota EL , Thompson SR , O’Bryan PJ , Cotta MA , Balan V , Jin M , Sousa L da C , Dale BE . 2016. Comparative lipid production by oleaginous yeasts in hydrolyzates of lignocellulosic biomass and process strategy for high titers. Biotechnol Bioeng 113:1676–1690. doi:10.1002/bit.25928 26724417

[5] Pomraning KR , Collett JR , Kim J , Panisko EA , Culley DE , Dai Z , Deng S , Hofstad BA , Butcher MG , Magnuson JK . 2019. Transcriptomic analysis of the oleaginous yeast Lipomyces starkeyi during lipid accumulation on enzymatically treated corn stover hydrolysate. Biotechnol Biofuels 12:162. doi:10.1186/s13068-019-1510-z 31289462 PMC6593508

[6] Monteiro de Oliveira P , Aborneva D , Bonturi N , Lahtvee P-J . 2021. Screening and growth characterization of non-conventional yeasts in a hemicellulosic hydrolysate. Front Bioeng Biotechnol 9:659472. doi:10.3389/fbioe.2021.659472 33996782 PMC8116571

[7] Jagtap SS , Bedekar AA , Singh V , Jin Y-S , Rao CV . 2021. Metabolic engineering of the oleaginous yeast Yarrowia lipolytica Po1F for production of erythritol from glycerol. Biotechnol Biofuels 14:188. doi:10.1186/s13068-021-02039-0 34563235 PMC8466642

[8] Jagtap SS , Deewan A , Liu J-J , Walukiewicz HE , Yun EJ , Jin Y-S , Rao CV . 2021. Integrating transcriptomic and metabolomic analysis of the oleaginous yeast Rhodosporidium toruloides IFO0880 during growth under different carbon sources. Appl Microbiol Biotechnol 105:7411–7425. doi:10.1007/s00253-021-11549-8 34491401

[9] Kim KE , Peluso P , Babayan P , Yeadon PJ , Yu C , Fisher WW , Chin C-S , Rapicavoli NA , Rank DR , Li J , Catcheside DEA , Celniker SE , Phillippy AM , Bergman CM , Landolin JM . 2014. Long-read, whole-genome shotgun sequence data for five model organisms. Sci Data 1:140045. doi:10.1038/sdata.2014.45 25977796 PMC4365909

[10] Travers KJ , Chin C-S , Rank DR , Eid JS , Turner SW . 2010. A flexible and efficient template format for circular consensus sequencing and SNP detection. Nucleic Acids Res 38:e159. doi:10.1093/nar/gkq543 20571086 PMC2926623

[11] Bushnell B , Rood J , Singer E . 2017. Bbmerge – accurate paired shotgun read merging via overlap. PLoS One 12:e0185056. doi:10.1371/journal.pone.0185056 29073143 PMC5657622

[12] Grigoriev IV , Nikitin R , Haridas S , Kuo A , Ohm R , Otillar R , Riley R , Salamov A , Zhao X , Korzeniewski F , Smirnova T , Nordberg H , Dubchak I , Shabalov I . 2014. Mycocosm portal: gearing up for 1000 fungal genomes. Nucleic Acids Res 42:D699–704. doi:10.1093/nar/gkt1183 24297253 PMC3965089

[13] Kuo A , Bushnell B , Grigoriev IV . 2014. Fungal genomics: sequencing and annotation. In Advances in botanical research. Vol. 70. 10.1016/B978-0-12-397940-7.00001-X.

[14] Haridas S , Salamov A , Grigoriev IV . 2018. Fungal genome annotation. In: fungal genomics: methods and protocols. Methods Mol Biol 1775:171–184. doi:10.1007/978-1-4939-7804-5_15 29876818

